# Measuring the globalization of cities from the new regionalism perspective

**DOI:** 10.1186/s40064-016-3179-0

**Published:** 2016-09-13

**Authors:** Oylum Şehvez Ergüzel, Hakan Tunahan, Sinan Esen

**Affiliations:** Department of International Trade, Sakarya University, 54187 Serdivan, Sakarya Turkey

**Keywords:** Global competitiveness, New regionalism, Cities and countries, Trade performance, Trade outcomes indicators

## Abstract

The study aims to analyze the export performance of countries and of cities within them to identify synchronized or unsynchronized movement between them. In the empirical part of the study, the measurements used to analyze the export performance of the countries included in the literature are applied to establish the export performance of a single city—Sakarya, Turkey. These measurements include the Herfindahl–Hirchman product and market concentration indices, the Lawrence index, the trade complementarity index, and the Grubel–Lloyd intra-industry index, as well as additional indicators with local or regional contexts. The limited number of studies analyzing the export competitiveness of a single city with relevant formats in the literature reveal the significance of the study.

## Background

Cities are prominent worldwide because of the concentration of social and economic activities within them. Five billion people—60 % of the world’s population—will live in cities by 2030 so, they are the places where the most of the world population live and work. As the world’s economic engines, cities generate 80 % of gross domestic product (GDP) and 70 % of greenhouse gas emission (McKinsey Global Institute 2015). As a result, globalization itself now increasingly presents as an intercity phenomenon since it has been lowering national borders. This concept is compatible with *new regionalism*, which describes integration to a global system through microscale formations instead of macroscaled regions. Two questions concerning the increasing of cities in the global system arise: (a) Is it possible for developing countries to grow and integrate into global system through cities? (b) Is it possible for cities a different economic structure and unsynchronized path with countries? The first question refers to synchronized economic movement between city and country. Also, cities emerge as units that supporting countries to be parts of global system as a whole. This question also contradicts the concept of new regionalism because it gives more importance to countries as global actors; the latter defines cities as the main, sovereign actors in a global system—a definition which is compatible with new regionalism.

To answer these questions, basic economic indicators (e.g., job creation, productivity, economic growth, and export performance) should be compared among countries and the cities within them. *Export performance*, which measures externalities of cities’ and countries’ export potential, is an especially important indicator; it reveals trade performance, competitiveness, products, potential markets, as well as high-tech production processes and export-based developmental processes. Using this framework, the study analyzes cities’ and countries’ export performance to determine the synchronized or unsynchronized movement between them.

Every new global development leads to new tendencies toward polarization in the world. As a result of this polarization, the concept of new regionalism has emerged. New regionalism describes of the process of integration with a global system through microscale transactions, rather than though actions across macroscaled regions.

From the perspective of new regionalism, cities and clusters have come into particular prominence. Today, cities such as New York, Tokyo, London, Hong Kong, and Istanbul have integrated into the global system to a greater extent than the countries that contain them, albeit on a microscale. Because of their employment opportunities, exported goods, and social effects, such cities have emerged as new players in a globalized world. Trade affects the process of integration of cities and clusters into the globalized system; therefore, subnational regionalism has become as significant for development and competition as supranational regionalism.

Advanced technologies and increased mobility has opened the way to globalization and this started changing the structure of international relations. Global access to information and alternative sources has created a new form of organizing—and, thus, governing—particular regions around the world, which, due to globalization, have become politically and economically more significant than others (Scott and Storper 2003).

Cities are the natural outcome of rapid globalization; they have the capability to both compete globally and benefit from globalization. (Mommas 2004).

Cities unite various types of productive activities from across regions and sectors, and, therefore play a major role in the economic and political arena of a globalized world. Because their formation and existence depends on strong economic and political links related to the regions within which they exist, cities have become much more important than traditional, geographically defined territories.

This study aims to analyze and compare the export performance of particular countries and their sovereign cities in order to identify synchronized or unsynchronized movement between them. The first part of the study examines the general export structure and performance of Turkey and Sakarya, an influential export city in Turkey.

### The foreign trade structure of Turkey and Sakarya

**Fig. 1:**
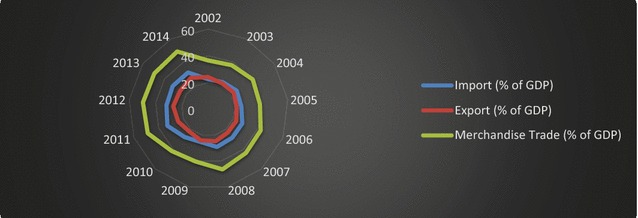
Turkey’s merchandise trade, import, and export (% of GDP). *Source*: World Bank (2014)

Its young population, strong banking sector, and geographical location have contributed to Turkey’s status as an actor and a market in the globalized world. Additionally, foreign trade, in interaction with the abovementioned factors, occupies a crucial role in the Turkish development processes. Figure 1 illustrates Turkish merchandise trade, import, and export as a share of GDP.

Turkey’s merchandise trade increased during the global economic crisis of 2008. In 2009, the real impact of the crisis was observed, and Turkey’s economy shrank by 4.7 %. The result of this shrinkage can be seen as a drop in Fig. 1. Total industrial production, which constitutes one-fourth of national GDP, experienced a marked decline after the second quarter of 2008; this decline triggered an economic contraction nationwide (Ertuğrul et al. 2010).

Technologically intensive production is a crucial indicator of competitiveness in the world economy. Table 1 shows Turkey’s exports based on technological intensity during 2013 and 2014.

**Table 1:** Export of Turkey based on technology intensity. *Source*: TSI (Turkish Statistical Institute)

	2013		2014	
	Value^a^	(%)	Value^a^	(%)
Total manufacturing industries	141,358	100	147,158	100
High-technology industries	4789	3.4	5020	3.4
Medium–high-technology industries	44,540	31.5	46,517	31.6
Medium–low-technology industries	43,329	30.7	42,984	29.2
Low-technology industries	48,700	34.5	52,635	35.8

Classification of product group by technology intensity is prepared by the Organization of Economic Cooperation and Development (OECD) based on ISIC. Rev. 3 classification

^a^Based on FOB value

Turkey has increased its medium-technology exports since the 1980s, while its high-technology exports have remained stagnant. The share of medium-technology exports—as measured by total exports—increased by half of the last decade, while high-technology exports could not gain a foothold in the export basket (TSI 2014). In addition, since the 1980s, Turkey has increased its medium-technology exports while high-technology exports have remained low. Moreover, despite improvements in medium-technology exports, the quality ranking of Turkish exports remained low, especially in European Union (EU) markets (World Bank 2014).

Turkey’s low export ranking can partially be attributed to the relatively low level of foreign direct investment (FDI) in the country’s manufacturing sector; as a result of globalized production, increasing the flow of FDI has provided positive spillover through productivity. Foreign-owned firms tend to be more productive, and predominantly high-technology and skill-based, as compared to domestically owned companies. For these reasons, rising shares of FDI in Turkey’s manufacturing sector will increase product quality and diversification while catalyzing the production of technologically advanced goods. As a result, Turkey must shift to the production and trade of more high-income goods and services, and move up the value chain in sectors in which it is already specialized.

**Fig. 2:**
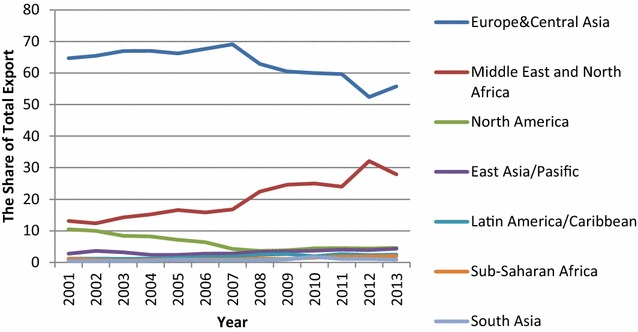
Turkey’s exports by region as a share of total exports, 2001–2013. *Source*: TSI (2015)

Due to the global financial crisis, free trade agreements, diplomatic measures, and the economic shrinkage of European Markets, Turkey’s export and import markets have changed to sufficiently avoid the negative effects of that period. The changing composition of Turkey’s export and import market is represented in Fig. 2.

**Fig. 3:**
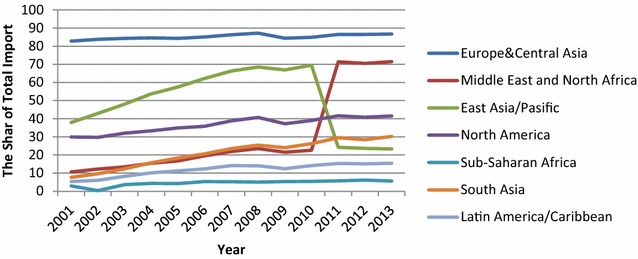
Turkey’s imports by region as a share of total exports, 2001–2013. *Source*: TSI (2015)

Turkey’s market diversification with respect to particular regions can be analyzed according to Figs. 2 and 3. Figures show that over the past decade, the European Union has remained an important trading partner for Turkey. However, Middle East and North African (MENA) countries have gained more prominence, while export shares of European Union countries have diminished since 2008. During the economic crisis, the fall in exports to the EU-27 and the United States began earlier, lasted longer, and was more significant than decreases in exports to all other regions. Meanwhile, a long-term decline in the U.S. market continued between 2007 and 2010; the share of exports to the United States declined to 3.3 %. However, this is associated with the end of the Multi Fibre Arrangement among textile-producing countries, rather than with the financial crisis (World Bank 2014). As a result, although European Union countries remain the most important trade partners for Turkey, Turkish products have appeared in new markets. This diversification toward nontraditional markets—particularly at a time when demand of the EU decreased—benefits the country. Moreover, diversification in product composition provides an increased level of sophistication. Turkey has especially gained comparative advantage in new products, such as road vehicles, compared to many of its peers, including Brazil, Russia, India, China, and South Africa (BRICS).

An important aspect of Turkish foreign trade and economic growth is the performance of the nation’s cities, which have been engines of economic growth. Therefore, analyzing the export performances of cities represents an important indicator of Turkey’s level of integration into a global system.

With a population of 1 million, Sakarya is located between Ankara and Istanbul. Of 81 cities in Turkey, Sakarya is ranked ninth in Turkish exports; furthermore, of the top ten Turkish export cities of 2014, Sakarya has seen the greatest increase in its exports (Turkish Exporters Assembly 2015). Additionally, it has an increased ability to compete within and integrate into a global system. As a city in Turkey, Sakarya has the economic potential to be a strong regional and global actor. Table 2 shows Sakarya’s exports, imports, and foreign trade volume between the years 2002 and 2014.

**Table 2:** Foreign trade of Sakarya (2002–2014). *Source*: TSI (2015)

Year	Exports (millions of dollars)	Imports (millions of dollars)	Foreign trade volume (millions of dollars)
2002	428,029	527,905	955,934
2003	843,017	751,905	1,594,923
2004	2,093,254	1,193,818	3,287,071
2005	2,712,960	1,555,407	4,268,367
2006	2,981,394	1,930,986	4,912,380
2007	3,522,655	2,018,569	5,541,224
2008	2,912,889	1,708,866	4,621,755
2009	1,722,375	908,949	2,631,324
2010	1,678,285	1,005,238	2,683,523
2011	2,011,778	1,368,469	3,380,247
2012	1,820,384	1,149,585	2,969,969
2013	2,250,874	1,639,155	3,890,028
2014	2,599,044	1,663,822	4,262,866

**Fig. 4:**
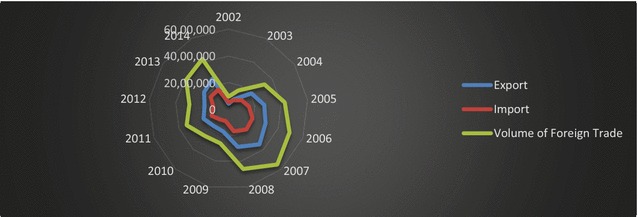
Foreign trade of Sakarya (2002–2014). *Source*: TSI (2015)

As Fig. 4 indicates, Sakarya’s foreign trade increased from 2002 to 2008, but experienced a severe decrease during the next 3 years, with the effect of the Global Financial Crisis. Although the progress seen in 2011 did not continue in 2012, economic recovery occurred in 2013 and 2014. Nevertheless, Sakarya still couldn’t reestablish its pre-2008 export values.

**Fig. 5:**
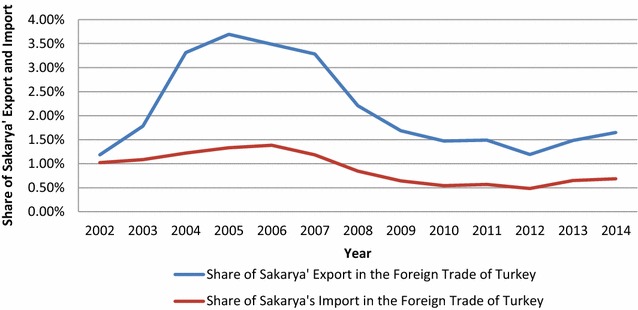
Share of Sakarya in total foreign trade of Turkey (2002–2014). *Source*: Data obtained from TSI (2015); calculated by the authors

**Fig. 6:**
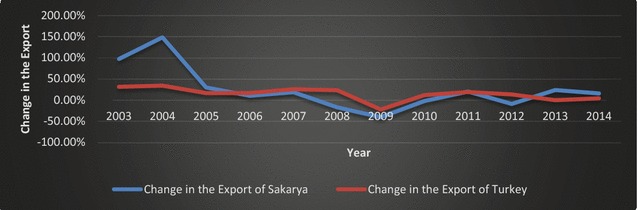
Changes in the exports of Sakarya and Turkey (2002–2014). *Source*: TSI (2015); calculated by the authors

**Fig. 7:**
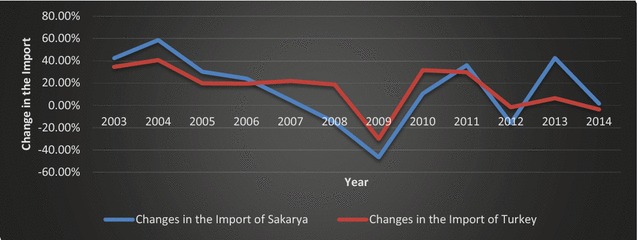
Changes in the imports of Sakarya and Turkey (2002–2014). *Source*: TSI (2015); calculated by the authors

Figure 5 shows the change in the share of Sakarya’s foreign trade per annum. The graph indicates that—especially until the Global Financial Crisis—Sakarya’s share in Turkey’s exports fell in the 3–3.5 % range, despite the insignificant declines; however Sakarya’s exports weathered a considerable decline with the crisis. Despite its later economic recovery, Sakarya’s exports constituted only 1.65 % of Turkey’s exports at the end of 2014. In contrast, Turkish imports did not experience a similar decrease during a similar period. Sakarya’s imports constitute about 1 % of Turkey’s foreign trade; this ratio is 0.69 % as of the end of 2014. As the data indicates, movements seen in Sakarya’s foreign trade seem to originate with the city’s internal dynamics. Figures 6 and 7 illustrate the movement of Sakarya’s foreign trade in relation to Turkey’s foreign trade.

As indicated by Figs. 6 and 7, the global financial crisis had a greater impact on the export and imports of Sakarya than on Turkey overall. The difference can be attributed to a contraction in the automotive sector, which constitutes a significant portion of Sakarya’s exports.

**Fig. 8:**
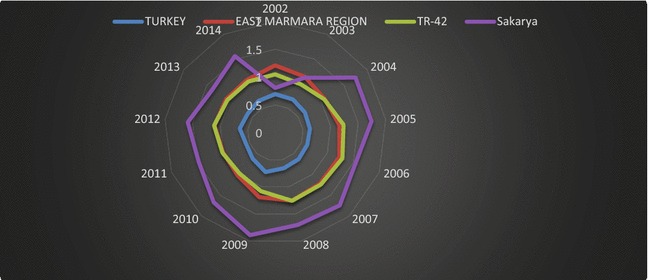
Comparative regional trade performance of Sakarya and Turkey. *Source*: TSI (2015); calculated by the authors

According to Nomenclature of Territorial Units for Statistics (NUTS), Turkey consists of 12 regions as a candidate for the European Union (TSI 2015). Sakarya is located in the East Marmara Region (TR4) with other developed, industrialized cities (e.g., Bursa, Kocaeli, and Eskisehir). Moreover, Sakarya is a part of the Kocaeli subregion (TR42), which consists of cities such as Kocaeli, Yalova, Düzce, and Bolu. In this context, Fig. 8 shows the comparative regional trade performances of Sakarya and of Turkey overall, which is computed by dividing total exports by total imports.

The trade performance of Sakarya exceeded the trade performance of Turkey during the period of 2002–2014. In general, Sakarya has demonstrated more favorable trade performance than the trade performance of East Marmara and TR42 regions. As the comparative regional trade performance analysis indicates, Sakarya has outperformed both the regions where it is positioned, as well as Turkey. The following section includes a review of literature on the measurement of export performance of macrounits.

### Review of literature

Exports constitute an important factor for both cities and countries due to the positive relationship between trade and growth. In addition, it is crucial that policy makers who want to benefit from the positive impacts of exporting on the improved productivity, decreasing unemployment, and accumulation of foreign exchange reserves (Sousa 2004, p. 15). Furthermore, one must account for the viability of the development and competition of production sectors of many countries in order to enter and sustain in global markets. For these reasons, the impact of export on different areas of the economy is among the most studied subjects in the literature.

Frenkel and Romer (1999), Panas and Vamvoukas (2002), Safdari et al. (2011), Waithe et al. (2011), Abbas (2012) and Fontoura and Crespo (2015) examine the relationship between export performance and economic growth; their studies confirm the positive relation that exists between two variables by using different statistical methods. Kraay (1999), Wagner (2007), Taymaz and Yılmaz (2007), Pisu (2008) and Cebeci (2014) analyze the relationship between productivity and export performance on the level of the firm’s level. Generally, trade performance indices are used to evaluate the connection between economic growth and export performance of a designated location. Trade outcome indicators—introduced by the World Bank—represent an effective tool with which to analyze export performance and economic growth, using different indices.

The literature suggests that a country or region’s export performance tends to be a good indicator of economic performance. However, it is difficult to establish a definition of successful trade performance. For example, some regions or countries record high export performance by concentrating on niche markets and specific products, while others show more moderate performance with well-diversified products and markets. In other cases, successful performance may be a result of an area’s ability to adapt its export profile to the changing patterns of world demand (International Trade Centre 2007). For these reasons, different metrics have been used to determine the different dimensions of export structure, including the orientation of export composition, the diversification of export patterns, and the sophistication of an export portfolio of a region or country.

Within this scope, studies exist that use indices to analyze the export performance of different countries and regions with respect to the diversification, sophistication, and concentration of their exports. These studies are explored in greater detail below.

*Export diversification* is defined as a transformation in the composition of an existing export portfolio or destination. Diversification provides stability in export earnings and a broader base of exports, thus enhancing economic growth. There are two important questions about diversification: (a) “Why do regions or countries diversify their exports?” and (b) “Do countries benefit from diversity in economic growth and development?” (Samen 2010). Export diversification can reduce volatility and instability in export earnings. This is widely accepted in principle (Derosa 1992). Specifically, Ghosh and Ostry (1994), Ramey and Ramey (1995), Bleaney and Greenaway (2001) and Reis and Farole (2013) indicate that more diversified economies were less vulnerable in terms of output, and lower output volatility. Concentration on a few products may have serious negative economic and political consequences. Insufficient diversification may lead to instability in foreign exchange earnings, which had negative macroeconomic impacts on growth, employment, investment planning, import and export capacity, foreign exchange cash flow, inflation, and debt repayment (Cashin and McDermott 2010). Political risks exist, especially, in countries that have suffered from civil wars or deteriorating governance. Due to the volatility of commodity prices, export-oriented developing countries suffered from economic, political, and social turmoil (Collier 2002). Moreover, limited diversification in primary and agricultural products increased vulnerability to external shocks, and thus interrupted growth due to terms of trade deterioration (Sarkar 1986). Export diversification aims to eliminate these negative economic and political results. In addition, sustained, rapid growth was found to be highly related to export growth (Brenton et al. 2009). Furthermore, rapid export growth was associated with diversification into new product markets. According to Bora et al. (2004), the exports of low-income and developing nations showed less-diversified export structures. Also, Imbs and Wacziarg (2003) and Rajagopal (2007) indicated that economies are inclined to diversify until they reach upper-middle income status. Moreover, specialization in some product categories is dominated within the export structure of developing and low-income countries.

The most frequently used measures of diversification are product and market concentration ratios, such as the Herfindahl and Hirschman Product and Market concentration Indices, The trade complementarity index, the Lawrence index (Lawrence 1984), and the Grubel and Lloyd Intra-Industry Trade Index. These measures are discussed in the following paragraphs.

The Herfindahl–Hirschman index (HHI), referred to as the “Herfindahl Index” is a measure used to evaluate both the product and market concentration of export; it is one of the most widely used—and criticized—measures used for such a purpose (Guordan 2010). The HHI was first used in the 1940s to measure skewness, and was formally adopted in economic theory in 1976 (Cowling and Waterson 1976). In 1984, the U.S. Department of Justice used the HHI as a concentration index for mergers; this application has been followed by many others for regulatory and academic purposes. Although the HHI has many commonly accepted uses, it has received wide criticism (Lijesen 2004). Tirole (1998) made the main criticism of the HHI, claiming that the index generally ignores important factors that affect and determine market powers, including costs of entry and asymmetries in costs and demand.

The trade complementary index is used to determine the compatibility of regional or national exports with imports of a potential partner country (Michaeley 1996). This index implies that both regions or countries gain from the trade partnership when one has a comparative advantage in products in which the partner has comparative disadvantage (World Integrated Trade Solution 2013).

Intra-industry trade is the mutual trade of products that fall under the same industry classification (Clark 2010). Balassa (1996) and Grubel and Lloyd (1975) analyzed trade of similar but differentiated products rather than specialization. In addition to this, Krugman (1979) and Lancester (1980) introduced a trade theory of monopolistic competition models based on assumptions of increasing return to scale and consumers’ love for variety. Grubel and Lloyd (1975) developed an empirical study to measure intra-industry trade. The most important advantage of intra-industry trade arises from its basic characteristics of economies of scale and decreasing costs. Intra-industry trade emerges from each country’s production of a limited range of products in the same industry. Economies of scale arise from specialization in different and differentiated products in the same sector. In this way, countries have decreased fixed costs and benefited from economies of scale, as well as provided an increased variety of goods for domestic consumers (Marrewijk 2009).

In addition to studies based on the export performance of different countries and regions, studies specifically about Turkey and its provinces have been increasingly prevalent in the literature. Yılmaz (2003), Akal (2008), Çeviker and Taş (2011), Özlale and Cunedioğlu (2011), Gros and Selçuki (2013) and Erkan (2014) have all examined Turkey’s export performance, comparing sales to different regions such as the EU and MENA by using different export performance indices to reveal the diversification, sophistication, and concentration patterns of Turkey’s export structure. In addition to these, the World Bank’s “Trading Up To High Income Report” (2014) analyzes the comparative advantage of Turkey’s exports by comparing it to Russia, Azerbaijan, China, and the MENA countries, which have been nontraditional trading partners with Turkey over the past decade.

In addition to these, some studies have analyzed the trade composition of different cities in different regions of Turkey.

The Economic Policy Research Foundation of Turkey (2010) investigated export performance and competitiveness potential with respect to the exports of 81 cities, categorized their respective technological classification of export, diversification of products and market, and ubiquity and trade complementarity. Sakarya ranks sixth in the study in terms of the export of high- and medium-technology products, while Kocaeli—a city located in the same region as Sakarya—ranks third. Additionally, *ubiquity* is a measurement used to evaluate the characteristics of exporting products. Products that are exported by many cities are defined as *ordinary products*, with a high ubiquity. Sakarya ranks third, after Istanbul and Rize, in respect to export ubiquity among all other cities of Turkey. Also, the Development Agency of East Marmara (2010), the Development Agency of the West Black Sea (2013), and the Development Agency of Ankara (2013) all analyzed the export performance of different regions and provinces of Turkey by using different export performance indices.

### Data and methodology

We intend to (a) expose cities’ and countries’ synchronized or unsynchronized economic movement, and (b) identify their capacity to integrate within a globalized world. In this study, analysis of export performance is used as a basic economic indicator. In particular, the country of Turkey and Sakarya, a city within Turkey, are analyzed to investigate this relationship.

A substantial portion of the data used in the study is obtained from the Turkish Statistical Institute (TSI) and the United Nations International Trade Centre Database. The analyses include the years 2002–2014 and 2002–2015 (when available), because the year 2002 marks the beginning of the foreign trade data of TSI for individual Turkish cities.

The remainder of the data is obtained from the database containing daily export information of all the exporters in Sakarya, except those registered in Akyazı—a district of Sakarya where is not included in Sakarya Chamber of Commerce and Industry. The data were collected with the cooperation of the Sakarya Chamber of Commerce and Industry and the Sakarya University International Trade Department. The database is composed of information gathered from Invoice, ATR, and Euro1 movement certificates, as well as the certificate of origin given to the Chamber by the exporter during export transactions.

The measurements are made using two protocols, when applicable: (a) the Harmonized Commodity Description and Coding System, generally known as the “Harmonized System” (HS); and (b) the United Nations International Standard Industrial Classification of all Economic Activities Revision 3.1 (ISIC). The HS is a multipurpose, international product nomenclature introduced by the World Customs Organization, used for measurements that are based on the exporting product. This system is used by more than 200 countries and economies as a basis for custom tariffs and the collection and analysis of international trade statistics. Over 98 % of merchandise traded internationally is classified according to the HS (World Customs Organization 2015). Meanwhile, the ISIC is used in analyses based on exporting sectors; this system represents the common international standard for the classification of economic activities. The aim of the system is to provide a standard set of economic activities. For this purpose, entities can be classified according to the activity they implement.

### Findings and discussion

In this part of the study, the Herfindahl–Hirschman (HHI) market and product concentration index, Lawrence index, trade complementarity index, and Grubel–Lloyd index are used to determine the performance and the structure of exports from both Turkey as a whole and Sakarya in particular.

#### Measurements of market and product concentration of Turkey and Sakarya

In this part of study, the Herfindahl and Hirschman market and product concentration indices are calculated and interpreted to determine market and product concentration for Turkey and Sakarya.

The *concentration ratio* (CR) for exporting markets is an indicator that expresses the cumulative shares of a certain number of countries. Table 3 shows the top ten export markets, and their concentration rates in relation to both Turkey and Sakarya.

**Table 3:** Top 10 export markets and their concentration rates for Turkey and Sakarya in selected years. *Source*: Data is obtained from TSI (2015); calculated by the authors

CR	2002			2006			2010			2014		
	Country	Share (%)	CR (%)	Country	Share (%)	CR (%)	Country	Share (%)	CR (%)	Country	Share (%)	CR (%)
*Turkey*												
CR1	Germany	16.2	16.2	Germany	11.3	11.32	Germany	10	10.0	Germany	9.61	9.6
CR2	USA	9.31	25.5	UK	7.9	19.29	UK	6.3	16.4	Iraq	6.91	16.5
CR3	UK	8.39	33.9	Italy	7.8	27.19	Italy	5.7	22.1	UK	6.28	22.0
CR4	Italy	6.59	40.5	USA	5.9	33.1	France	5.3	27.4	Italy	4.53	27.3
CR5	France	5.92	46.4	France	5.3	38.49	Iraq	5.3	32.7	France	4.1	31.4
CR6	Russia	3.25	49.7	Spain	4.3	42.83	Russia	4.0	36.8	USA	4.02	35.4
CR7	Spain	3.12	52.8	Russia	3.7	46.62	USA	3.3	40.1	Russia	3.77	39.2
CR8	Neth.	2.93	55.7	Iraq	3.0	49.65	Spain	3.1	43.2	Spain	3.01	42.2
CR9	Israel	2.39	58.1	Neth.	2.9	52.62	UAE	2.9	46.1	UAE	2.95	45.2
CR10	Belgium	1.9	60.0	Romania	2.7	55.3	Iran	2.6	48.4	Iran	2.4	47.6
*Sakarya*												
CR1	Israel	13.8	13.8	Germany	15.6	15.6	Germany	18.6	18.6	Russia	13.1	13.1
CR2	Germany	9.5	23.3	France	10.5	26.2	Spain	12.8	31.4	Israel	9.4	22.5
CR3	Finland	8.4	31.7	Spain	9	35.2	France	8.4	39.7	Germany	7.8	30.4
CR4	Ireland	7.5	39.2	Russia	6.7	41.9	UK	8.4	48.1	UK	7.5	37.9
CR5	Italy	7	46.3	UK	6.7	48.6	Sweden	5.9	54	France	6.6	44.5
CR6	Poland	5.8	52.1	Italy	5.1	53.7	Italy	4.8	58.8	Belgium	6.2	50.7
CR7	Russia	4.3	56.4	Finland	4.2	57.9	Belgium	4.1	62.9	Egypt	4.9	55.5
CR8	Azerb.	3.8	60.1	Belgium	4.1	62	Poland	3.7	66.6	Spain	4.7	60.3
CR9	Portugal	3.4	63.5	Neth.	4.1	66.1	Russia	3.6	70.2	Poland	4.6	64.9
CR10	Spain	2.5	66	Denmark	2.7	68.8	Switzer.	2.8	73	Sweden	4.3	69.1

According to Table 3, while diversification of the export market increased between 2002 and 2014 for Turkey, Sakarya’s export market composition has concentrated on a more limited number of markets during the same period.

Additionally, there was a decrease in concentration of Turkey’s export market structure. This indicates that although global trade slowed during the global economic crisis, Turkey minimized potential negative impacts by diversifying its export market with new countries and regions. Turkey compensated for decreasing shares of EU countries in its export market composition with increasing shares of MENA countries. As a result, the concentration rate of Turkey’s top ten export partners dropped from 61 in 2002 to 47.66 % in 2014. Furthermore, concentration increased in Sakarya’s exporting markets. In contrast with Turkey as a whole, Sakarya’s export composition concentrated on a more limited market between 2002 and 2014.

**Fig. 9:**
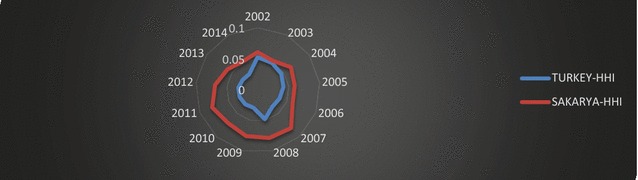
Herfindahl–Hirschman market concentration index of Turkey. *Source*: Data is obtained from TSI (2015); calculated by the authors

In this study, the Herfindahl–Hirschman (HH) market and product concentration indices are used to determine diversification in the export structure of Turkey and Sakarya. The Herfindahl–Hirschman market concentration index is an indicator of dependency on trading partners. An index value nearer to 1 indicates concentration and a high dependency on very few markets. Figure 9 shows the Herfindahl–Hirschman market concentration index results for Turkey and Sakarya.

As we see in Fig. 9, Turkey’s HH market concentration index was highest in 2008, during the global financial crisis. As mentioned, Turkey has diversified its export market to MENA countries to compensate for a decreasing share of European Union markets. This development led to a decrease in Turkey’s index value between 2009 and 2014, indicating that Turkey diversified its export market by trading with different countries, rather than concentrating on a few markets. In the specific case of Sakarya, dependence on the European market affected its index values. Sakarya had more diversified export market composition in 2014, with an index value of 0.05, while it had the most concentrated dependency on exporting markets in 2007, with an index value 0.08. It seems that Sakarya has increased the diversification of its export markets since 2011 to mitigate the negative impacts of the global crisis and decreasing European demand.

The Herfindahl–Hirschman product concentration index gives greater weight to the larger export categories; a value of unity indicates exports of only one commodity or service (high concentration), while diversification increases as index values approach 0.

In this context, diversification in export products and sectors of Turkey and Sakarya have been specified by using the Herfindahl–Hirschman product concentration index with respect to the ISIC Rev.3.1 sector and HS product classifications over the period 2002–2014.

The automotive sector in Sakarya constitutes roughly 69 % of its exports as a result of authors calculations. To determine the impacts of the automotive sector on diversification in the exporting sector of “Manufacture of Motor Vehicles,” 3410 ISIC. Rev. 3.1 and “HS87—Vehicles other than railway or tramway rolling-stock, and parts and accessories thereof” have been excluded from calculations. Table 4 shows the Herfindahl–Hirschman product concentration index values for export sectors of Turkey and Sakarya with respect to ISIC Rev.3 and HS2.

**Table 4:** Herfindahl–Hirschman product concentration index of Turkey and Sakarya. (ISIC. Rev.3/HS2). *Source*: Data is obtained from TSI (2015); calculated by the authors

Year	Turkey—ISIC Rev.3	Turkey—ISIC Rev.3 (except ISIC 3410)	Turkey—HS2	Turkey—HS (except HS87)	Sakarya—ISIC Rev.3 (except ISIC 3410)	Sakarya—ISIC Rev.3	Sakarya—HS2	Sakarya—HS (except HS87)
2002	0.050	0.053	0.043	0.045	0.112	0.473	0.521	0.196
2003	0.047	0.049	0.044	0.043	0.133	0.601	0.653	0.224
2004	0.047	0.046	0.047	0.042	0.194	0.782	0.801	0.275
2005	0.041	0.039	0.043	0.037	0.236	0.764	0.812	0.306
2006	0.039	0.035	0.044	0.037	0.296	0.742	0.817	0.302
2007	0.039	0.034	0.047	0.038	0.221	0.681	0.809	0.284
2008	0.042	0.039	0.048	0.042	0.102	0.653	0.815	0.103
2009	0.033	0.030	0.039	0.034	0.112	0.631	0.764	0.096
2010	0.031	0.039	0.038	0.033	0.074	0.596	0.692	0.075
2011	0.030	0.028	0.038	0.034	0.077	0.582	0.686	0.082
2012	0.033	0.034	0.04	0.039	0.079	0.394	0.537	0.08
2013	0.026	0.024	0.031	0.031	0.086	0.421	0.572	0.075
2014	0.025	0.023	0.030	0.029	0.102	0.475	0.651	0.076

As shown in Table 4, diversification in export sectors and products for Turkey increased considerably during the period of 2002–2014. However, export product diversification of Sakarya remained at almost at the same level throughout the same period.

Table 4 shows that for Turkey, exclusion of the automotive sector did not lead to major changes in index values. This suggests that Turkey’s dependency on the export pattern of the automotive sector is low, and that its exported sectors and products are well diversified.

In the case of Sakarya, authors see decreases in index values as compared to index values for all products. This indicates that concentration in exporting sectors has been caused by the Manufacture of Motor Vehicles. Index values for other products are close to 0, which indicates that other exporting sectors of Sakarya do not consist of a few, specified sectors. Conversely, calculations have been made by excluding the product category “Vehicles other than Railway or Tramway Rolling-Stock, and Parts and Accessories thereof,” using the HS.87 product code to determine diversification in other exported products of Sakarya. In this instance, decreasing index values indicated diversified characteristics in other exporting markets.

### The Lawrence index measurement for structural changes in export of Turkey and Sakarya

**Fig. 10:**
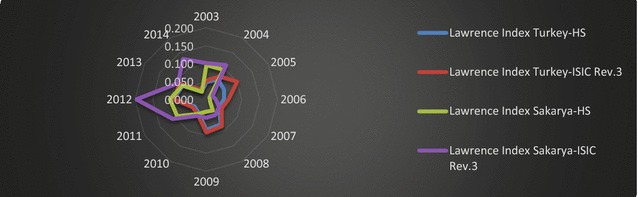
Lawrence indices of Sakarya and Turkey. *Source*: Data is obtained from TSI (2015); calculated by the authors

In this part of study, the authors implement the Lawrence index to present the structural changes in export patterns of Turkey and Sakarya. This index has been computed using annually based data to determine the detailed export structure of Turkey and Sakarya. Results have been presented in terms of ISIC Rev.3.1 and HS classifications in Fig. 10.

The values of the Lawrence index with respect to ISIC Rev. 3 and HS 2 classifications are very low during the years 2003 and 2014; this suggests that there was no important structural change for the export patterns of Turkey and Sakarya. Therefore, changes in the export patterns of Turkey or Sakarya are explained by cyclical factors (such as sectorial or global economic changes), which are dominated in the short and medium terms.

As indicated in Fig. 10, the higher value was observed in 2012 for Turkey and Sakarya between 2003 and 2014. To make a more accurate assessment of these changes, global events that affect demand for exported products should be considered as factors that impact the export structure of Sakarya and Turkey. According to the Trade and Development Report of the United Nations (2012), international trade expansion and a robust recovery in 2010 slowed to 5.5 % by 2012. Due to the 2008 financial crisis, weak demand, especially in the EU, was considered an important factor affecting those economies—like Turkey and Sakarya—which greatly depend on exports to EU countries. Therefore, a recession in these economies has direct impact on the export composition of Turkey and Sakarya.

Besides the global trade slowdown in 2012, the authors investigated changes in the main sectors to explain high index values. Examination of the automotive sector was a priority, given that the automotive industry is a main actor in Turkey’s manufacturing sector. Between 2000 and 2014, original equipment manufacturers (OEM) invested more than 12 billion USD operating in Turkey; as a result of this, their manufacturing capabilities increased tremendously. This phenomenon led Turkey to become an important part of the global value chain of international OEMs. In order to meet high international quality and safety standards (and due to value-added production), the modern Turkish automotive industry has become highly efficient and competitive. Turkey accounts for 25 % of automotive production occurring in Central and Eastern Europe. Furthermore, the automotive industry is one of the largest employers in the country, with a potential to create job opportunities for more than 400,000 people. With three out of five top exporters hailing from the automotive industry, the sector represents a substantial 16 % of total exports (Republic of Turkey Prime Ministry Investment Support and Promotion Agency 2015). In 2010, the Turkish automotive sector experienced a 10.03 % contraction compared to the previous year. In December 2010, Turkey experienced a 9.78 % decline in the passenger car and light commercial vehicles market, as compared with the same month of 2011.

**Fig. 11:**
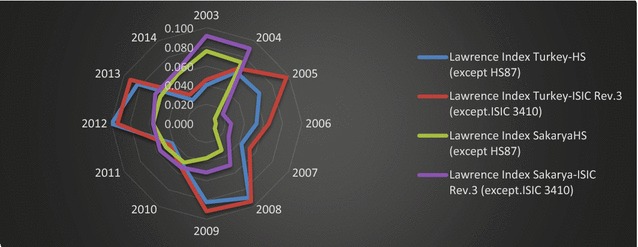
Lawrence indices of Sakarya and Turkey (except HS87 and ISIC Rev3). *Source*: Data is obtained from TSI (2015); calculated by the authors

To understand the impact of contraction in the automotive sector on the trade performance of Sakarya and Turkey, index values have been recalculated by excluding automotive sectors and products in respect of ISIC Rev.3.1 and HS2 classifications; the results are shown in Fig. 11.

The contraction in automotive sectors had a significant impact on the export pattern of Sakarya, as indicated by the decreasing index values. When authors exclude the automotive sector, though, the index value in 2012 returns, approximately, to its levels in 2010 and 2011. However, excluding the automotive sector has not led to major changes in the index values.

### Trade complementarity and Grubel–Lloyd indexes of Turkey and Sakarya

In this part, authors apply trade complementarity and Grubel–Lloyd indices to reveal the export patterns of Turkey and Sakarya in detail.

#### Trade complementarity index

The similarity between the export basket of exporters and import profile of trade partners affects whether both an exporter and importer will benefit from increased trade. In this case, one must consider the extent to which the export pattern of the exporter matches the import profile of the trade partner. A higher index result indicates a higher complementarity value, as well as a better export/import match; a value of 0 indicates no complementarity at all. In this way, levels of complementarity are determined between composition of Turkey and Sakarya in relation to the import pattern of the 192 countries in the world. Table 5 shows the countries that have the strongest trade complementarity with the export profiles of Turkey and Sakarya.

**Table 5:** Top 10 countries with strong trade complementarity. *Source*: Data is obtained from World Trade Organization (2015); calculated by the authors

2010—Turkey		2011—Turkey		2012—Turkey		2013—Turkey		2014—Turkey	
Country	Index	Country	Index	Country	Index	Country	Index	Country	Index
Iraq	73.38	Iraq	75.35	Iraq	75.57	Iraq	79.23	Luxembourg	66.41
Luxembourg	68.92	Luxembourg	68.38	Iran	69.79	Myanmar	70.93	Saudi Arabia	65.80
Sudan	68.27	Austria	67.24	Saudi Arabia	65.07	Iran	67.09	Austria	65.64
Iran	67.32	Slovenia	66.68	Luxembourg	64.96	Austria	66.61	Bahrain	65.34
Slovenia	65.86	Latvia	65.1	Austria	64.36	UAE	65.75	Canada	64.93
Austria	65.41	Canada	64.72	Georgia	64.21	Luxembourg	65.28	Romania	64.13
Portugal	65.23	Slovakia	64.29	Macedonia	63.19	Georgia	65.11	Norway	64.07
Canada	64.50	Georgia	64.25	Slovenia	63.16	Norway	65.09	Qatar	64.05
Kuwait	63.67	Norway	64.03	Kuwait	63.13	Canada	64.82	Algeria	63.62
Uzbekistan	62.95	Macedonia	62.13	Canada	62.39	Kuwait	64.74	Georgia	57.11

2010—Sakarya		2011—Sakarya		2012—Sakarya		2013—Sakarya		2014—Sakarya	
Country	Index	Country	Index	Country	Index	Country	Index	Country	Index
Oman	35.85	Oman	40.08	Oman	40.15	Nepal	50.02	Kuwait	35.71
Nigeria	33.82	Nigeria	35.21	Argentina	37.32	Oman	40.95	Saudi Arabia	33.69
Zimbabwe	31.52	Argentina	31.87	Saint Hel.	36.73	Bahrain	38.86	Ghana	32.41
Argentina	33.11	Saudi Arabia	29.58	Russia	36.37	Nigeria	37.47	Qatar	31.84
Canada	27.59	Canada	29.56	Canada	36.34	Ghana	36.55	Bahrain	31.84
Brunei D.	27.46	Bolivia	28.98	Uzbekistan	34.24	Argentina	36.4	Oman	31.04
Luxembourg	27.22	Saint Hel.	28.22	Zimbabwe	33.93	Saudi Arabia	35.72	Canada	29.88
Portugal	26.85	Slovakia	27.56	Slovakia	33.26	Suriname	35.2	Slovenia	29.79
Uzbekistan	26.45	Libya	27.47	Lux.	33.12	Qatar	34.56	Libya	28.94
Australia	25.87	Russia	27.28	Austria	32.41	Canada	33.95	Algeria	28.13

According to Table 5, Iraq has the most sustainable strong trade complementarity with Turkey between the 2010 and 2013. Luxembourg is ranked second, followed by Saudi Arabia, Austria, Canada, Kuwait, and Norway. In contrast to other years, 2014 indicates that MENA countries such as Algeria, Qatar, and Bahrain have strong trade complementarity with Turkey. Kuwait, Saudi Arabia, Bahrain, Oman, and Libya have strong complementarity with Sakarya. Table 6 shows that the countries have increased trade complementarity with Turkey and Sakarya.

**Table 6:** Increasing trade complementarity with Turkey and Sakarya. *Source*: Data is obtained from World Trade Organization (2015); calculated by the authors

Turkey						Sakarya					
Countries/year	2010	2011	2012	2013	2014	Countries/year	2010	2011	2012	2013	2014
UAE	60.4	60.8	62.49	65.09	64.07	Saudi Arabia	19.84	29.59	28.74	35.73	33.69
Qatar	52.82	61.36	62.34	60.44	64.05	Bahrain	21.67	20.79	25.10	37.06	31.84
Belarus	54.75	54.31	52.66	59.25	58.21	Kuwait	31.20	32.09	38.03	41.34	35.71
Azerbaijan	56.84	53.95	55.78	58.90	62.23	UAE	17.23	21.82	21.70	26.28	26.43
Bahrain	48.53	48.18	47.04	56.95	65.34	Qatar	19.91	25.65	26.29	34.56	31.84
Bulgaria	57.75	58.01	55.97	59.02	61.06	Serbia	18.94	22.63	29.63	32.48	26.46
Algeria	58.14	57.66	57.88	63.40	63.63	Slovenia	26.27	26.59	32.12	29.87	29.79
Israel	55.57	54.32	60.44	57.68	62.35	Turkmenistan	21.66	23.49	27.40	27.85	30.56
USA	55.93	56.67	56.56	58.96	59.05	Germany	22.04	23.77	29.58	27.19	24.15
Czech Republic	59.55	62.33	60.77	62.83	62.49	USA	22.79	23.58	28.36	27.62	24.89

Table 6 shows that countries have the most strong trade complementarity with the export profile of Turkey and Sakarya. As seen in the table, trade complementarity between most MENA countries and Turkey has increased between 2010 and 2014. Accordingly, EU countries represent the most crucial trading partners for Turkey and Sakarya. However, the weight of MENA countries in export baskets of Turkey and Sakarya increased after the global financial crisis. These results match with those of the Herfindahl–Hirschman Market Concentration Index. To mitigate the negative impacts of the global financial crisis and attendant decrease in demand from European countries, diversification has been inevitable in Turkey and Sakarya. The trade complementarity index indicates the direction of export market diversification; therefore, Turkey and Sakarya have shifted and diversified their export markets from European countries to MENA regions.

#### Grubel–Lloyd intra-industry trade

**Fig. 12:**
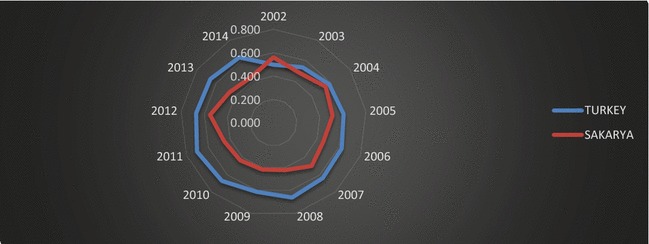
Adjusted Grubel–Lloyd intra-industry trade index. *Source*: Data is obtained from TSI (2015); calculated by the authors

Intra-industry trade analyses examine trade of similar but differentiated products, rather than specialized products. Figure 12 illustrates the adjusted Grubel–Lloyd intra-industry trade index for Turkey and Sakarya over the period of 2002–2014.

As seen in Fig. 12, Turkey’s intra-industry trade increased after crisis periods such as 2002 and 2009. During this 12-year period, Turkey exported differentiated products in different sectors. However, Turkey’s intra-industry trade pattern increased between 2002 and 2014. This means that Turkey has increasingly exchanged similar products belonging to the same industry. Figure 12 suggests that intra-industry trade is generally low for Sakarya; it has the higher values in 2002 and 2012.

## Conclusion

The most remarkable aspect of cities is their economic competitiveness, which arises from their potential for trade, increasing population, clustered technology, industry, and employment.

Globalization has led to the increasing interdependence of economies through trade, global financial markets, information systems, technology, and production. The most important result of global interdependence is an increasing competitiveness among global actors. Due to increasing population, economic activities, and employment potential, cities have emerged as strong actors in the new globalized economy, based on the competition of said actors. In this context, cities’ relationships with the countries in which they are sited represent controversial phenomena. There are two options for the location of cities on a global level; they either act as (a) complementary components of countries, or (b) main, sovereign actors in global system. While the former contradicts the new regionalism theory, the other latter does not.

Within this context, the study aims to analyze cities’ and countries’ export performances to identify synchronized or unsynchronized movement between them and compare their competitiveness potential using indices and measurements. The Herfindahl–Hirschman market concentration index indicates that Turkey diversified its export market by exchanging with different countries so as to not to concentrate solely on a few markets. Conversely, Sakarya has increased its diversification in export markets since 2011 to mitigate the negative impacts of the global crisis and corresponding decreases in European demand. The Herfindahl–Hirchman product concentration index shows that diversification in Turkey’s export sectors and products increased considerably between 2002 and 2014. The study also shows that the dependency of Turkey’s export pattern on the automotive sector is very low, and that exported sectors and products are well-diversified. In contrast, export product diversification in Sakarya has remained at almost at the same level during the same period. However, the concentration level decreases when manufacture of motor vehicles is excluded from the analysis.

The values of the Lawrence index with respect to ISIC Rev. 3 and HS 2 classifications have been very low between 2003 and 2014; this indicates an absence of significant structural change to the export pattern of either Turkey and Sakarya. This means that changes in the export patterns of Turkey and Sakarya can be explained by cyclical factors, such as sectorial or global economic change, which is dominated in the short and medium terms.

Levels of complementarity are determined by comparing the export composition of Turkey and Sakarya with the import patterns of the 192 countries in the world. EU countries remain the most crucial trading partners for both Turkey and Sakarya. However, the weights of various MENA countries in the export baskets of Sakarya and Turkey have increased in the aftermath of the global financial crisis. These results match with those of the Herfindahl–Hirschman market concentration index.

Turkey’s intra-industry trade pattern increased between 2002 and 2014; this indicates that Turkey has increasingly exchanged similar products that belong to the same industry. However, intra-industry trade remains generally low for Sakarya. Therefore, increases in index values regarding exporting markets can be attributed to global crises and decreasing demand from European markets. Index values show that the export pattern of Sakarya has been more affected by sectorial and market changes that correspond with the city’s limited market and its high dependency on the automotive industry. Changes in export markets have had a substantial impact on the export performance of Turkey due to high product diversification. Compared with adjacent regions, and with Turkey as a whole, Sakarya’s export performance makes it a strong actor.

Technology intensity definitions of manufacturing industries of OECD define the automotive industry—an important sector in the manufacturing industry—as a medium- to high-technology sector group. This sector represents an opportunity for Sakarya’s export composition to create externalities and increase diversification with more value-added sectors; this will increase potential employment, trade, population, technology, industry, and economic activities, as well as making Sakarya a strong actor by increasing its global competitiveness. Sakarya’s export concentration on a single sector with a few companies indicates that institutional authorities should support diversification of both products and markets. Furthermore, regional agencies and policy makers should support the potential of Sakarya on a sustainability basis, given that this study indicates that Sakarya has comparative advantages in regards to its region and Turkey.

## Abbreviations

ATR: European-Turkey movement certificate
BRICS: Brazil, Russia, India, China and South Africa
CR: concentration ratio
EU: European Union
FDI: foreign direct investment
GDP: gross domestic product
HHI: Herfindahl–Hirschman index
HS: Harmonized System
ISIC Rev. 3.1: The United Nations international standard industrial classification of all economic activities revision 3.1
MENA: Middle East and North Africa
NUTS: Nomenclature of Territorial Units for Statistics
OEM: original equipment manufacturers
OECD: Organization of Economic Cooperation and Development
TSI: Turkish Statistical Institute
TR4: East Marmara Region of Turkey
TR42: one of the subregion of Turkey where consists of Sakarya, Kocaleli, Yalova, Düzce and Bolu as cities
US: United States
WITS: World Integrated Trade Solution
